# Quantitative analysis of lacewing larvae over more than 100 million years reveals a complex pattern of loss of morphological diversity

**DOI:** 10.1038/s41598-023-32103-8

**Published:** 2023-04-14

**Authors:** Carolin Haug, Florian Braig, Joachim T. Haug

**Affiliations:** 1 Ludwig-Maximilians-Universität München (LMU Munich), Großhaderner Str. 2, 82152 Planegg-Martinsried, Germany; 2 GeoBio-Center at LMU, Richard-Wagner-Str. 10, 80333 München, Germany

**Keywords:** Developmental biology, Evolution, Zoology

## Abstract

Loss of biodiversity and especially insect decline are widely recognised in modern ecosystems. This decline has an enormous impact due to the crucial ecological roles of insects as well as their economic relevance. For comparison, the fossil record can provide important insights on past biodiversity losses. One group of insects, for which a significant decline over the last 100 million years has often been postulated, but not demonstrated quantitatively, is Neuroptera (lacewings). Many adult lacewings are pollinators, while the larvae are mostly predators, which becomes very obvious from their prominent stylet-like mouthparts. We investigated the fossil record of larvae of all neuropteran lineages as well as a large share of extant neuropteran larvae. Based on these, we performed an outline analysis of the head with stylets. This analysis provides a quantitative frame for recognising the decline of lacewings since the Cretaceous, indicating also a severe loss of ecological roles.

## Introduction

The loss of diversity in the group Insecta is now widely recognised^1,2^, also by the broader public (e.g.^3^). Losses of biodiversity in the past, as documented by the fossil record, are thought to be informative to improve our understanding of the general processes leading to such losses (e.g.,^4–7^).

To recognise losses, changes in biodiversity need to be assessed accurately. This assessment is especially challenging when comparing diversity in the past to modern diversity. For example: Neuroptera, the group of lacewings, is generally accepted to have been part of the early diversification of Holometabola (wasps, flies, and all their closer relatives) and having been much more diverse in the past than it is today^8^. However, this supposed loss of lacewing diversity is not easy to demonstrate quantitatively. The modern fauna includes more than 6000 species of lacewings, but only about 1000 fossil species have been recognised so far^9^. Therefore, species numbers alone do not reflect a case of diversity loss.

Diversity loss could also be investigated at higher taxonomic levels, e.g. ranks such as “family”. However, these higher taxonomic ranks are somewhat arbitrary, as objective criteria when a certain taxonomic rank should be given to a specific monophyletic group are lacking (e.g.^10–12^). Even the number of families of lacewings in the modern fauna is a matter of debate and differs between authors: 19^13^, 17–18^8^, 16^9,14–16^, 15^17,18^ or 14^19^; overall 21 names for supposed families can be found (references above;^20^). Some newer publications have reduced the numbers by 1 (recent example in^21^), but others have revived long synonymised names^22^ and even introduced new ones^23,24^. The resulting range in the numbers of families in the modern fauna (≈13 to 21) is quite large and depends on the taxonomic interpretation of the author(s). Therefore, counting higher taxonomic units is unlikely to represent a reliable tool for recognising a loss of diversity in the present case as well.

In consequence, it seems that the loss of biodiversity in lacewings is perceived in a different way. What has been recognised appears to be a loss of morphologically distinct forms, which indicate that lacewings in the past fulfilled specific ecological functions that are nowadays no longer performed by their successors. Potentially, these roles are nowadays performed by representatives of other, now more diverse groups, such as butterflies^25^. Hence, the loss of diversity in lacewings is apparently not recognised via taxonomic diversity (related to species richness), but instead via morphological diversity (≈ disparity).

Part of the success of the large group Holometabola (including Neuroptera) has been attributed to the differentiation of ecological function between the adults and their larvae. Therefore, morphological diversity assessments should not be restricted to adults, but need to include larval stages as well. The larvae of lacewings are mostly fierce predators; each lower and upper jaw together form a forward-projecting stylet, which is used to inject venom and digestive fluids into the prey and suck the dissolved body tissues out. Lacewing larvae indeed show a large morphological variation in the modern fauna^8,17^, but even more so in the past, especially in the Cretaceous, as documented by different inclusions in amber of 130–100 million years of age (e.g.^26–30^).

Recent quantitative analyses of the morphologies of larvae and their changes over time in individual lineages of Neuroptera have revealed losses of morphological diversity in some lineages^28,31^, but not in all of these^29,32,33^. Here, we present the first comprehensive quantitative analysis of morphological diversity of the heads and stylets (mouthparts) of larvae of the entire group of Neuroptera over the last 130 million years, based on a dataset of more than 1,000 specimens. Of these, 230 specimens came from Cretaceous ambers (ca. 130–90 mya), 34 from Eocene ambers (ca. 40–35 mya), 12 from Miocene ambers (25–15 mya), and 787 from the modern fauna, in total 1063 larvae.

## Results

### Comparing diversity of morphological groups through time

Ideally, we would compare the diversity for each node along the phylogenetic tree of Neuroptera. However, there are certain challenges to this approach. First, uncertainties concerning the relationships of different ingroups still remain, for example, Ascalaphidae, the group of owlflies, may represent an ingroup of Myrmeleontidae, the group of antlions, or vice versa^21,27^. Yet, the monophyly of a group including Ascalaphidae and Myrmeleontidae is beyond doubt (hence collectively referred to as “owllions”^34^).

More challenging is especially the fact that we cannot reliably identify the closer relationships of many of the fossil larvae. We therefore need to compare the diversity for larger groups that in some cases do not represent natural (monophyletic) groups, but rather share an ecological role or represent functional groups, e.g. aphidlions (see discussion in^29^), sometimes with a shared distinct morphology such as larvae with straight stylets in Mantispoidea and Dilaridae^33^. Fossils can be identified as aphidlions or as possessing straight jaws^30^ and can then be compared to their modern counterparts.

After an initial analysis we excluded larvae of dustywings (Coniopterygidae). Their head shapes strongly differ from those of the other larvae (more or less triangular) and therefore strongly polarise the overall morphospace, disguising differences among the other larvae. Also so far there is no clear larva of Coniopterygidae in the Cretaceous, making the comparison more challenging. Coniopterygidae has been resolved as sister group to all other lacewings^9,14,15^, the comparison in the following therefore concentrates on the sister group to Coniopterygidae, Euneuroptera, the true lacewings^14^.

### Clear losses of diversity through time

**Figure 1:**
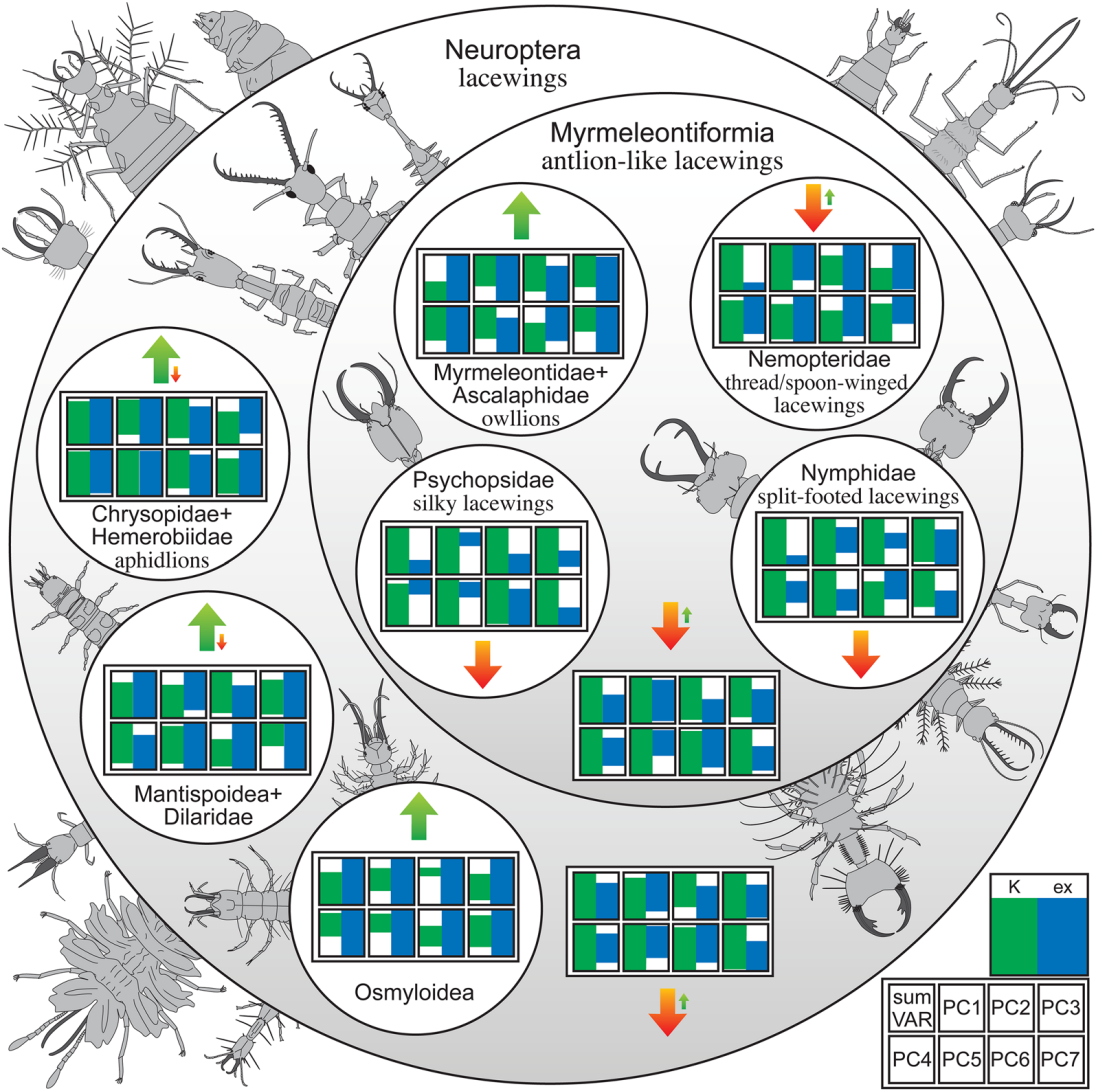
Simplified diversity changes of lacewing larvae in the last 130 million years; relative relationships simplified as Venn diagram. The overall changes of diversity for the different lacewing groups are presented as arrows, with green arrows pointing to diversity increase, orange arrows pointing to diversity decrease; small arrows indicating a diverging diversity change in an ingroup in contrast to the larger group. The boxes indicate the range for the total sum of variance of the bootstrapped and rare-fractioned data sets, and for each principal component separately of the untreated data sets. In total, the diversity of lacewing larvae has decreased (orange arrow at the bottom). Sketches show simplified morphologies of different representatives based on various sources; drawings produced by the authors. *ex* extant, *K* Cretaceous, *PC* principal component, *sum VAR* sum of variance. More differentiated plot with four time slices in Suppl. Fig. 1.

Of the four recognisable ingroups of Myrmeleontiformia, the group of antlion-like lacewings, three show a significant loss of larval diversity since the Cretaceous (Welch's two sample t-test, p-value < 0.001 for all comparisons), only the larvae of owllions seem to have diversified after the Cretaceous (Fig. 1; Suppl. Fig. 1; Suppl. Text 1), as already indicated^34^. Yet, in the Cretaceous there were numerous now extinct early offshoots of the larger group of antlion-like lacewings (Myrmeleontiformia^26,35–38^) with heads and mouthparts to a certain extent comparable to those of modern owllion larvae; these similarities are detectable, for example, in bearing prominent teeth or in similar values in some of the principal components (PCs; especially PC1, but also PC2; Fig. 1). During the Cretaceous, the lineage of split-footed lacewings (Nymphidae) was more diverse and seems to have occupied some areas of the morphospace that are in the modern fauna occupied by owllions (Fig. 1; indicated in^34^). The diversification of owllions therefore fills some of the space previously occupied by split-footed lacewings, but not all of it. In consequence, the group Myrmeleontiformia has lost morphological diversity of larvae since the Cretaceous, despite the later diversification of owllions.

The larvae of the three other functional groups in Neuroptera (Osmyloidea, aphidlions, and the straight-jawed larvae of Dilaridae and Mantispoidea) clearly have further diversified after the Cretaceous (Fig. 1). Also, in some lacewing groups the larvae most likely possessed still more plesiomorphic traits in the Cretaceous, lacking the characteristic features of their modern counterparts. Therefore, larvae of these lineages could not yet be identified. Example groups are Sisyridae and Ithonidae.

Despite all these diversifications, the overall diversity of the entire group Neuroptera has decreased after the Cretaceous (Fig. 1). Again, this loss relates to larvae that are not representatives of modern lineages. Extinct supersting larvae^36,37^ have similarities with some modern larvae of Osmyloidea^33^ and may have been replaced by them; others seem to have no clear equivalent in the modern fauna.

### Beyond the diversity loss

**Figure 2:**
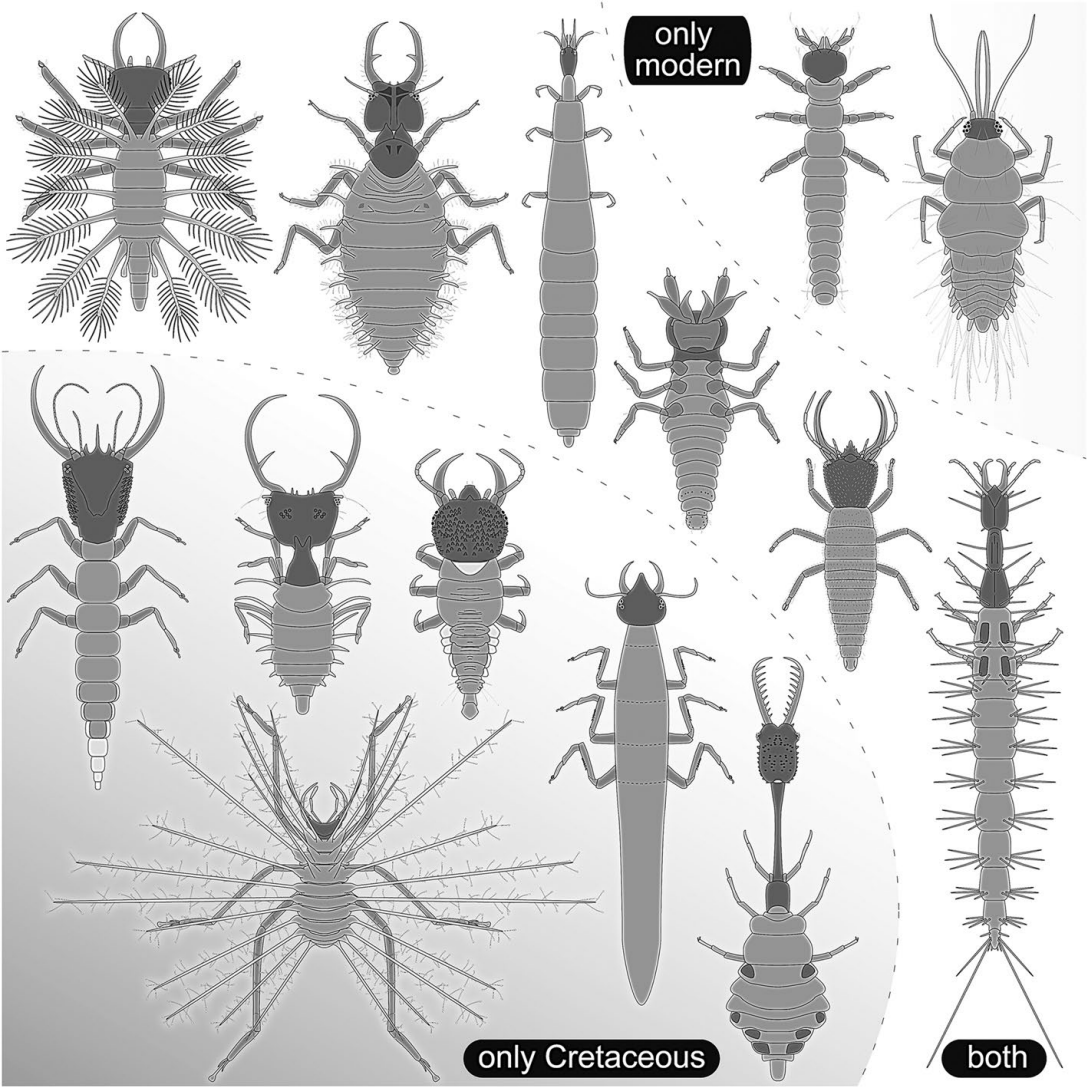
Overview on morphological diversity of lacewing larvae from the Cretaceous to today, illustrated with selected larval morphologies. Several morphologies are only known from the Cretaceous (left part), a certain number is known in the Cretaceous and the modern fauna (middle), while relatively few are only known in the modern fauna. Drawings based on various sources and produced by the authors.

Besides the fact that several morphologies of lacewing larvae have been lost since the Cretaceous, many morphologies have persisted since then (Fig. 2), indicated by a strong overlap in morphospace occupation. In addition, the modern fauna includes only few morphologies that have not yet been present in the Cretaceous. Most obvious examples are the modern larvae of Sisyridae (spongilla flies) with their extremely long stylets, which are used to parasitise on sponges, and the straight-jawed larvae of modern mantis lacewings (Mantispidae). These morphologies, only present today, are likely the result of diversification events after the Cretaceous, representing gains in diversity.

## Discussion

### Limitations of the approach

The comparison performed here remains asymmetric due to the nature of the fossil record, with a less clear signal from the less productive outcrops (Eocene and Miocene ambers). However, although our view back in time is limited to a smaller sample size and to very specific regions of the world, we can still recognise the larger morphological diversity of lacewing larvae in the Cretaceous. Taking biases into account, it is likely that the actual morphological diversity in the past was even larger than indicated by the current data.

The approach used here will likely only pick up strongly expressed cases of loss, as it might be not very sensitive due to the asymmetry of the sampling bias. For the sub-samples of the Miocene and Eocene, the (mostly) smaller diversity compared to the modern faunas is probably an artefact caused by the small sub-sample sizes. Cases in which a smaller sub-sample reveals a larger diversity (as for the Cretaceous) than the modern sub-sample likely represent a true signal. One might argue that the Cretaceous summarises a diversity of a longer time span. Yet in fact the vast majority of the Cretaceous samples (and therefore also the diversity) originates from a single locality, Kachin amber (Suppl. Table 1).

It proves to be important not only to compare fossils within their closely related lineages, but also in the wider frame^14^^, p. 545^. Some of the persisting morphologies (present in the past and today) may represent surviving old morphologies as in larvae of dragon lacewings (Nevrorthidae), yet others may represent cases of convergent evolution in which new evolving morphologies in one lineage replace extinct ones from another lineage^34^. Such more complex patterns will only be recognisable in larger-scaled analyses, as the one performed here.

### Overall a complex pattern, but a net loss of diversity

The approach applied here reveals an overall loss of morphological diversity in lacewings in a quantitative frame, but also reveals more complex aspects of it. While some lineages within Neuroptera undergo a decline, other lineages have diversified, partly “taking over”, or better convergently evolved some of the extinct morphologies and likely also ecological functions. We can also recognise that some modern-appearing fossil larvae, that can be identified as representing modern lineages, differ from their modern counterparts and represent extinct or replaced morphologies as well.

The results presented here can for the first time support the generally accepted loss of diversity in lacewings by quantitative and statistically supported measures. Furthermore it demonstrates that larvae can be well used for making such comparisons.

Given the variety of ecological functions which holometabolan larvae perform, the habit of excluding them from diversity studies is unfortunate. Quantitative morphology offers not only a tool for including larvae into a comparative frame as demonstrated here, it can also identify changes in diversity that can not be picked up quantitatively in a taxonomic frame.

## Methods

### Key resources table

**Table:** 

Reagent or resource	Source	Identifier
Deposited data		
Shapes and templates for shapes	Details in Suppl. Table 1	N/A
Software and algorithms		
R software-environment ver. 4.1.2	R Core Team 2021^39^	RRID:SCR_001905; https://cran.r-project.org/
Custom R scripts for analysis and statistical tests	This study	https://github.com/rianbreak/Neuroptera_1000.git
SHAPE	Iwata and Ukai 2002^40^	http://lbm.ab.a.u-tokyo.ac.jp/~iwata/shape/

### Quantification and statistical analysis

The data basis for this study were the outlines from earlier studies (see Key resources table; Suppl. Table 1; Suppl. Text 2). The biological shape was quantified by elliptic Fourier analysis (EFA), which applies the principle of the Fourier transformation to translate the two-dimensional outline into a mathematical object. We achieved this with the SHAPE software package^40^. The outline is translated into harmonics describing the shape^41^; we used 20 harmonics. The normalized elliptic Fourier descriptors (NEFDs) representing the specimens were aligned automatically based on the first harmonic. The results were analysed with a principal component analysis (PCA). The resulting principal components (PCs) were then used as input data for further statistical analysis and graphical interpretation. For further details, see Suppl. Text 1 and 3 and Suppl. Files 1–6.

All further analyses were performed offline using custom scripts in the R-statistics environment (ver. 4.1.0^39^). For initial visualization of the morphospace, PCs were plotted against each other in scatterplots, using ggplot2 (ver. 3.3.5^42^). We then calculated the range of values each ingroup of Neuroptera occupied for each PC, respectively for each time subset. Lastly, we calculated the sum of variances for each ingroup of Neuroptera, comparing the time subsets, using the package dispRity (ver. 1.6.0^43^; Suppl. Table 2; Suppl. Fig. 2). For this approach, we first bootstrapped each data set 10,000 times and applied rare-faction-based correction for differences in sample sizes. We used the sample size of the smaller group (often the fossil group) for this correction. We then tested the groups for significant differences in the sum of variance metric, using Bonferroni corrected pairwise Welch two sample t-tests.

## Supplementary Information

Supplementary Figure 1.

Supplementary Figure 2.

Supplementary Information 1.

Supplementary Legends.

Supplementary Table 1.

Supplementary Table 2.

Supplementary Information 2.

Supplementary Information 3.

Supplementary Information 4.

## Data Availability

This paper analyzes existing, publicly available data, listed in Suppl. Table 1. All data reported in this paper will be shared by the lead contact upon request. Joachim T. Haug (joachim.haug@palaeo-evo-devo.info).

## Code availability

All original code has been deposited at GitHub and is publicly available as of the date of publication. A link to the repository is listed in the key resources table. Any additional information required to reanalyze the data reported in this paper is available from the lead contact upon request.

## Material availability

This study did not generate new unique reagents.
