# Supplementary figures and images for: Quantitative analysis of lacewing larvae over more than 100 million years reveals a complex pattern of loss of morphological diversity
Source: Sci Rep. 2023 Apr 14;13:6127. doi: 10.1038/s41598-023-32103-8 (PMC10104811; doi:10.1038/s41598-023-32103-8)

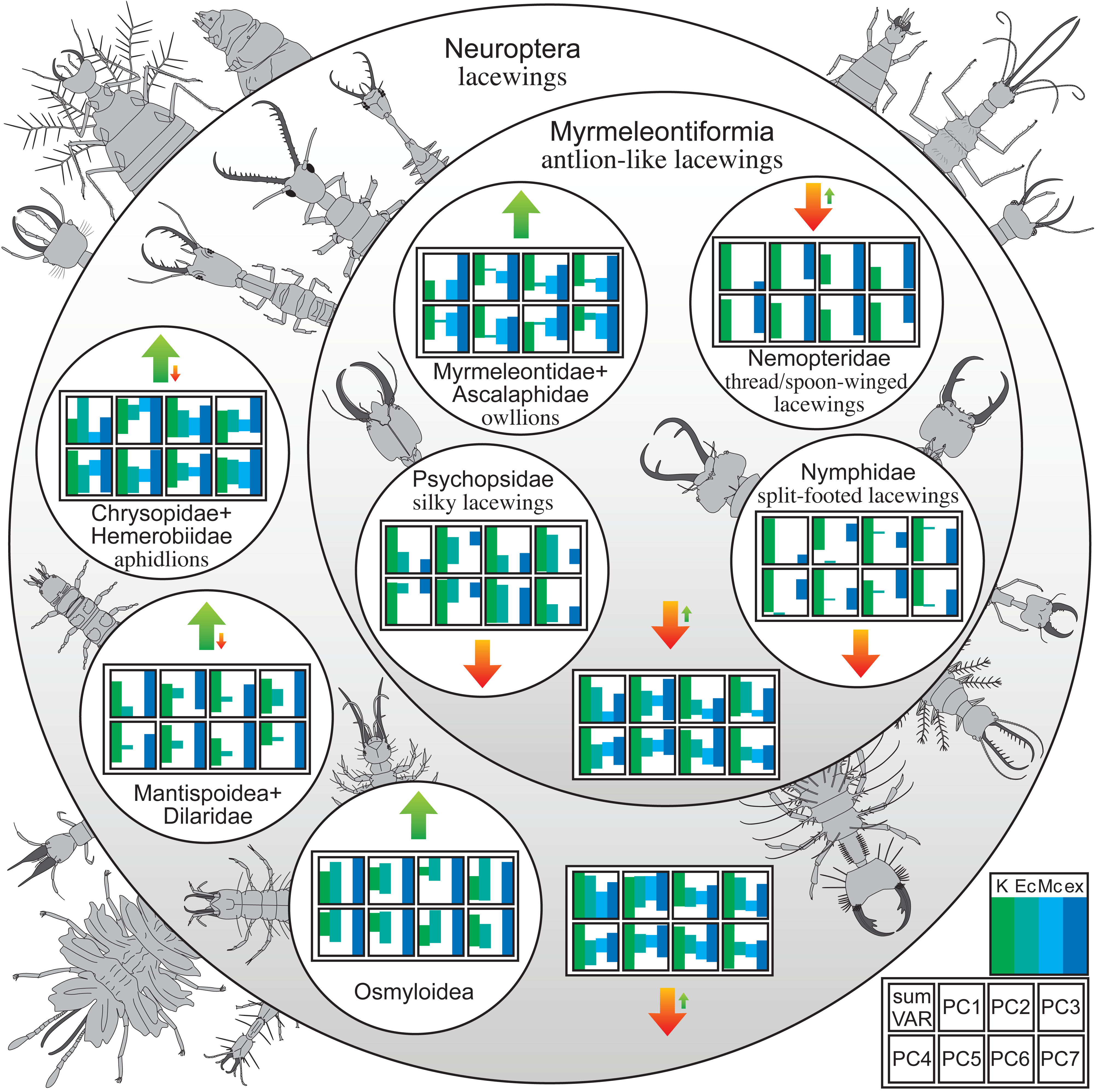

Supplement: Supplementary file 1 — Supplementary Figure 1. [file 41598_2023_32103_MOESM1_ESM.tif]

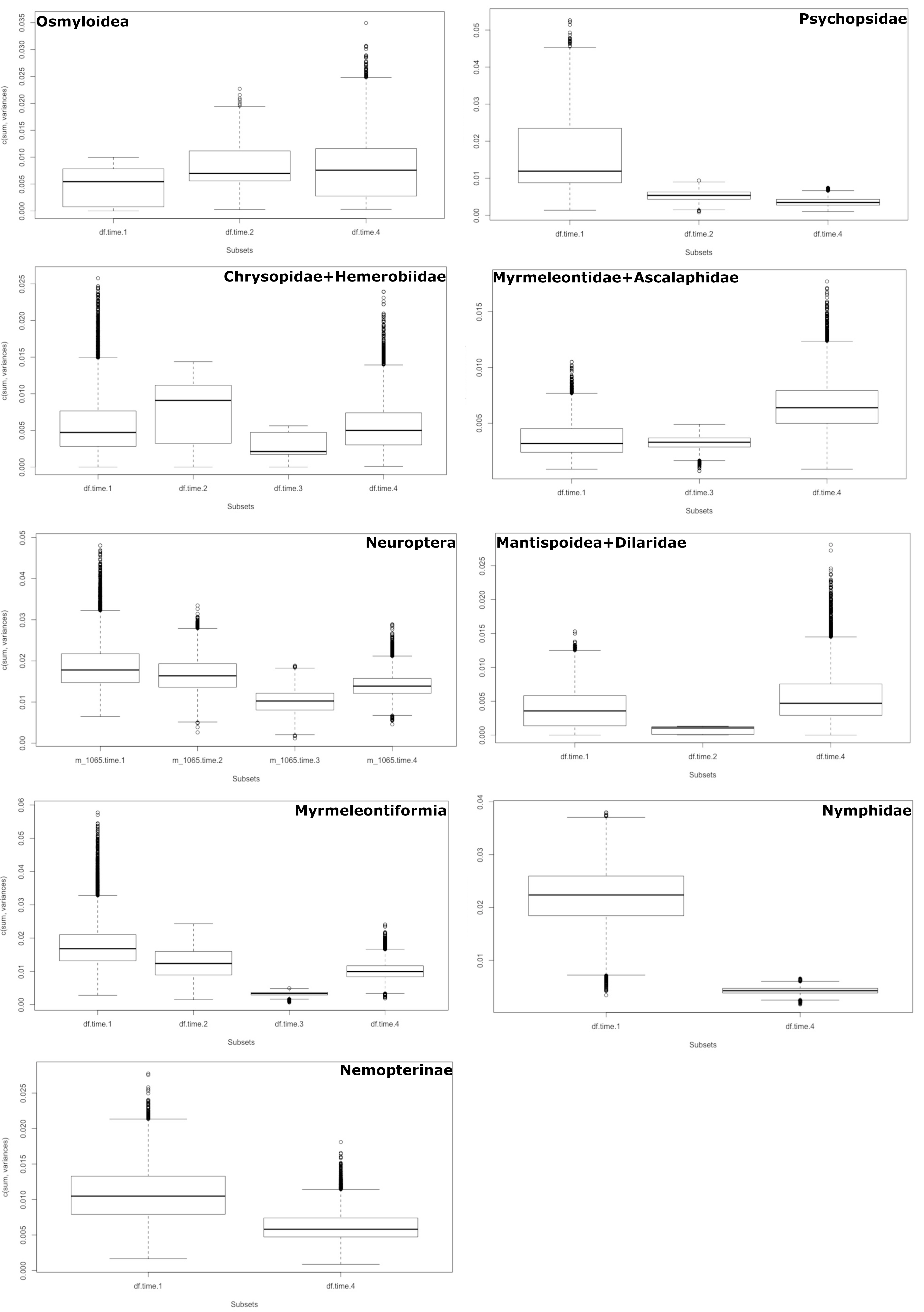

Supplement: Supplementary file 2 — Supplementary Figure 2. [file 41598_2023_32103_MOESM2_ESM.png]

-2S.D.

Mean

+2S.D.

PC1

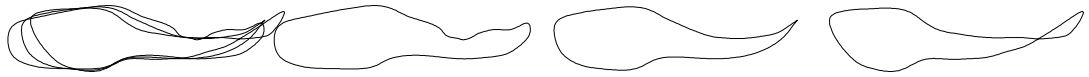

PC2

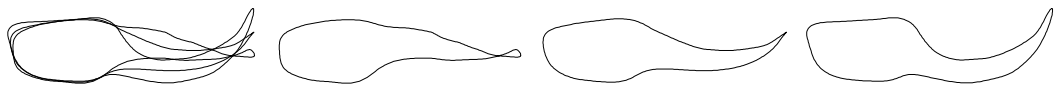

PC3

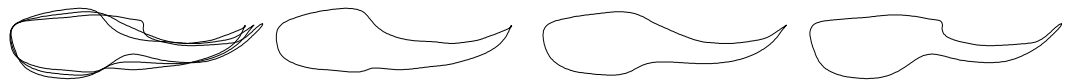

PC4

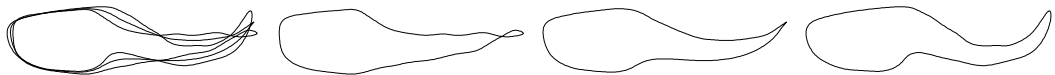

PC5

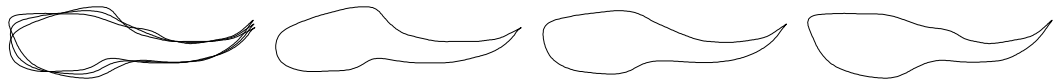

-2S.D.

Mean

+2S.D.

PC6

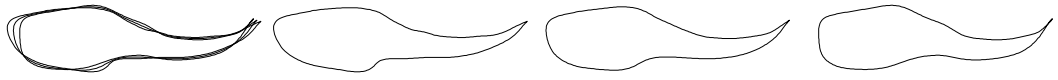

PC7

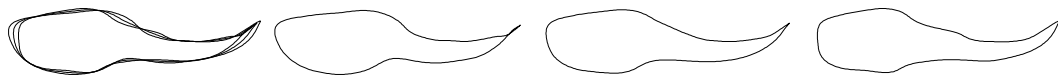

Supplement: Supplementary file 3 — Supplementary Information 1. [file 41598_2023_32103_MOESM3_ESM.zip › Suppl_files1-6_1000_Neuro/head_stylets_factor.pdf]
