# Supplementary material for: Quantitative analysis of lacewing larvae over more than 100 million years reveals a complex pattern of loss of morphological diversity
Source: Sci Rep. 2023 Apr 14;13:6127. doi: 10.1038/s41598-023-32103-8 (PMC10104811; doi:10.1038/s41598-023-32103-8)
Supplement: Supplementary file 4 — Supplementary Legends. [file 41598_2023_32103_MOESM4_ESM.doc]

**Legends of supplementary information:**

**Suppl. Fig. 1.** More detailed version of the diversity changes of lacewing larvae from Fig. 1 with four time slices instead of two. For detailed explanation of the figure, see caption of Fig. 1. Abbreviations: Ec = Eocene; ex = extant; K = Cretaceous; Mc = Miocene; PC = principal component; sum VAR = sum of variance.

**Suppl. Fig. 2.** Boxplots of disparity analyses of all compared groups, including all respective time slices.

**Suppl. Tab. 1.** Information on the specimens included in the analyses. Abbreviations: Eoc = Eocene; K = Cretaceous; Mio = Miocene.

**Suppl. Tab. 2.** Numerical values of boxplots of the disparity analyses of all compared groups, including all respective time slices.

**Suppl. Text 1:** Results of the shape analysis and occupation of morphospace.

**Suppl. Text 2:** References used in Suppl. Tab. 1.

**Suppl. Text 3:** Results of the principal component analysis of the shapes.

**Suppl. Files 1–6:** Files resulting from the shape analysis, including the graphical representation of the factor loadings of the shape analysis, chain codes, aligned shapes, and principal component analysis.
