# Supplementary material for: Quantitative analysis of lacewing larvae over more than 100 million years reveals a complex pattern of loss of morphological diversity
Source: Sci Rep. 2023 Apr 14;13:6127. doi: 10.1038/s41598-023-32103-8 (PMC10104811; doi:10.1038/s41598-023-32103-8)
Supplement: Supplementary file 7 — Supplementary Information 2. [file 41598_2023_32103_MOESM7_ESM.doc]

**Supplementary Text 1:**

*Results of the shape analysis*

The dataset included 1,063 individual shapes (Suppl. Tab. 1; Suppl. Text 2). The analysis resulted in seven effective principal components (PCs) (Suppl. Text 3; Suppl. Files 1–6).

PC1 explains 36.24% of the overall variation. It is dominated by the relative length of head capsule vs. stylet. A low value indicates a long head capsule and a shorter stylet, a high value indicates a shorter head capsule and a longer stylet.

PC2 explains 25.06% of the overall variation. It is dominated by the curvature of the stylet and the position of insertion on the head capsule. A low value indicates a straight stylet inserting very medially, a high value indicates a curved stylet inserting rather laterally.

PC3 explains 11.03% of the overall variation. It is dominated by the relative size of the stylet and the presence of a projecting labrum. A low value indicates a very prominent stylet and no projecting labrum, a high value indicates a less prominent stylet and a projecting labrum.

PC4 explains 8.92% of the overall variation. It is dominated by the curvature of the stylet and the position of insertion on the head capsule. A low value indicates a straight stylet inserting rather laterally, a high value indicates a curved stylet inserting far medially.

PC5 explains 4.54% of the overall variation. It is dominated by the shape of the head capsule. A low value indicates a head capsule with a concave posterior border, a high value indicates one with a convex posterior end.

PC6 explains 2.35% of the overall variation. It is dominated by the shape of the head capsule. A low value indicates a head capsule with a projecting antero-lateral edge, a high value indicates no such projection.

PC7 explains 2.11% of the overall variation. It is dominated by the relative length of head capsule vs. stylet. A low value indicates a long head capsule and a shorter stylet, a high value indicates a shorter head capsule and a longer stylet.

*Occupation of morphospace*

We evaluated the morphospace occupation of each group as a measure for the morphological diversity of the group. Hereby, we used two different measures for quantification: the realized ranges of each group across each PC of the untreated data sets, as well as the sum of variances of each group across all PCs. For the latter metric, the data was first bootstrapped and rare-faction corrected for differences in sample size. Then, we used pairwise, Bonferroni corrected, Welch’s two sample t-tests, to compare the sum of variance measures of a group between its different time slices.

When analysing all representatives of Neuroptera, the total morphospace occupation as measured by the sum of variances decreased from the Cretaceous to the modern fauna (Welch’s two sample t-test, p-value < 0.001;Fig. 1; Suppl. Figs. 1, 2; Suppl. Tab. 2). The same is the case for almost all PCs, Cretaceous representatives of Neuroptera occupy larger ranges than modern ones (Fig. 1; Suppl. Fig. 1; Suppl. Text 3).

When analysing the representatives of Osmyloidea, the total morphospace occupation strongly increased from the Cretaceous to the modern fauna (Welch’s two sample t-test, p-value < 0.001; Fig. 1; Suppl. Figs. 1, 2; Suppl. Tab. 2). The same is the case for almost all PCs, especially PC2. Modern representatives of Osmyloidea occupy larger ranges than fossil ones (Fig. 1; Suppl. Fig. 1; Suppl. Text 3).

When analysing the representatives of Mantispoidea and Dilaridae, the total morphospace occupation increased from the Cretaceous to the modern fauna (Welch’s two sample t-test, p-value < 0.001; Fig. 1; Suppl. Figs. 1, 2; Suppl. Tab. 2). The same is the case for most PCs, except PC2 and PC4, for which morphospace occupation actually decreased (Fig. 1; Suppl. Fig. 1; Suppl. Text 3).

When analysing aphidlions, the total morphospace occupation increased from the Cretaceous to the modern fauna (Welch’s two sample t-test, p-value < 0.001; Fig. 1; Suppl. Figs. 1, 2; Suppl. Tab. 2). The same is the case for almost all PCs, except PC2 and PC6, for which a slight decrease in morphospace occupation occurs (Fig. 1; Suppl. Fig. 1; Suppl. Text 3).

When analysing the representatives of Myrmeleontiformia, the total morphospace occupation again decreased from the Cretaceous to the modern fauna (Welch’s two sample t-test, p-value < 0.001; Fig. 1; Suppl. Figs. 1, 2; Suppl. Tab. 2). The same is the case for almost all PCs, except PC6, which shows a slight increase for the morphospace occupation of modern forms (Fig. 1; Suppl. Fig. 1; Suppl. Text 3).

When analysing owllions, the total morphospace occupation strongly increased from the Cretaceous to the modern fauna (Welch’s two sample t-test, p-value < 0.001; Fig. 1; Suppl. Figs. 1, 2; Suppl. Tab. 2). The same is the case for all individual PCs (Fig. 1; Suppl. Fig. 1; Suppl. Text 3).

When analysing silky lacewings, the total morphospace occupation strongly decreased from the Cretaceous to the modern fauna (Welch’s two sample t-test, p-value < 0.001; Fig. 1; Suppl. Figs. 1, 2; Suppl. Tab. 2). The same is the case for all individual PCs (Fig. 1; Suppl. Fig. 1; Suppl. Text 3).

When analysing thread/spoon-winged lacewings, the total morphospace occupation strongly decreased from the Cretaceous to the modern fauna (Welch’s two sample t-test, p-value < 0.001; Fig. 1; Suppl. Figs. 1, 2; Suppl. Tab. 2). The same is the case for almost all PCs, except PC7, which shows a slight increase (Fig. 1; Suppl. Fig. 1; Suppl. Text 3).

When analysing split-footed lacewings, the total morphospace occupation strongly increased from the Cretaceous to the modern fauna (Welch’s two sample t-test, p-value < 0.001; Fig. 1; Suppl. Figs. 1, 2; Suppl. Tab. 2). The same is the case for all individual PCs (Fig. 1; Suppl. Fig. 1; Suppl. Text 3).
