# Supplementary material for: Quantitative analysis of lacewing larvae over more than 100 million years reveals a complex pattern of loss of morphological diversity
Source: Sci Rep. 2023 Apr 14;13:6127. doi: 10.1038/s41598-023-32103-8 (PMC10104811; doi:10.1038/s41598-023-32103-8)
Supplement: Supplementary file 8 — Supplementary Information 3. [file 41598_2023_32103_MOESM8_ESM.doc]

**References for Supplementary Table 1:**

Froggatt, W.W. 1907. Australian Insects. William Brooks and Company, Sydney, Australia.

Haug, C., Herrera Flórez, A.F., Müller, P. & Haug, J.T. 2019a. Cretaceous chimera – an unusual 100-million-year old neuropteran larva from the “experimental phase” of insect evolution. Palaeodiversity 12, 1–11.

Haug, C., Haug, G.T., Baranov, V.A., Solórzano-Kraemer, M.M. & Haug, J.T. 2021d. An owlfly larva preserved in Mexican amber and the Miocene record of lacewing larvae. Boletín de la Sociedad Geológica Mexicana 73, A271220.

Haug, C., Posada Zuluaga, V., Zippel, A., Braig, F., Müller, P., Gröhn, C., Weiterschan, T., Wunderlich, J., Haug, G.T. & Haug, J.T. 2022c. The morphological diversity of antlion larvae and their closest relatives over 100 million years. Insects 13, 587.

Haug, G.T., Haug, C., Pazinato, P.G., Braig, F., Perrichot, V., Gröhn, C., Müller, P. & Haug, J.T. 2020a. The decline of silky lacewings and morphological diversity of long-nosed antlion larvae through time. Palaeontologia Electronica 23(2), a39.

Haug, G.T., Baranov, V., Wizen, G., Pazinato, P.G., Müller, P., Haug, C. & Haug, J.T. 2021a. The morphological diversity of long-necked lacewing larvae (Neuroptera: Myrmeleontiformia). Bulletin of Geosciences 96, 431–457.

Haug, G.T., Haug, C. & Haug J.T. 2021b. The morphological diversity of spoon-winged lacewing larvae and the first possible fossils from 99 million-year-old Kachin amber, Myanmar. Palaeodiversity 14, 133–152.

Haug, G.T., Haug, C., van der Wal, S., Müller, P. & Haug, J.T. 2022a. Split-footed lacewings declined over time: indications from the morphological diversity of their antlion-like larvae. PalZ 96, 29–50.

Haug, J.T., Müller, P. & Haug, C. 2019b. A 100-million-year old slim insectan predator with massive venom-injecting stylets – a new type of neuropteran larva from Burmese amber. Bulletin of Geosciences 94, 431–440.

Haug, J.T., Müller, P. & Haug, C. 2019c. A 100-million-year old predator: a fossil neuropteran larva with unusually elongated mouthparts. Zoological Letters 5, art. 29.

Haug, J.T., Baranov, V., Schädel, M., Müller, P., Gröhn, C. & Haug, C. 2020b. Challenges for understanding lacewings: how to deal with the incomplete data from extant and fossil larvae of Nevrorthidae? (Neuroptera). Fragmenta entomologica 52, 137–167.

Haug, J.T., Pazinato, P.G., Haug, G.T. & Haug, C. 2020c. Yet another unusual new type of lacewing larva preserved in 100-million-year old amber from Myanmar. Rivista Italiana di Paleontologia e Stratigrafia 126, 821–832.

Haug, J.T., Schädel, M., Baranov, V.A. & Haug, C. 2020d. An unusual 100-million-year old holometabolan larva with a piercing mouth cone. PeerJ 8, e8661.

Haug, J.T., Baranov, V., Müller, P. & Haug, C. 2021c. New extreme morphologies as exemplified by 100 million-year-old lacewing larvae. Scientific Reports 11, 20432.

Haug, J.T., Haug, G.T., Zippel, A., van der Wal, S., Müller, P., Gröhn, C., Wunderlich, J., Hoffeins, C., Hoffeins, H.-W. & Haug, C. 2021e. Changes in the morphological diversity of larvae of lance lacewings, mantis lacewings and their closer relatives over 100 million years. Insects 12, art. 860.

Haug, J.T., Linhart, S., Haug, G.T., Gröhn, C., Hoffeins, C., Hoffeins, H.-W., Müller, P., Weiterschan, T., Wunderlich, J. & Haug, C. 2022b. The diversity of aphidlion-like larvae over the last 130 million years. Insects 13, 336.

Haug, J.T., van der Wal, S., Gröhn, C., Hoffeins, C., Hoffeins, H.-W. & Haug, C. 2022e. Diversity and fossil record of larvae of three groups of lacewings with unusual ecology and functional morphology: Ithonidae, Coniopterygidae and Sisyridae. Palaeontologia Electronica 25, a14.

Haug, J.T., Kiesmüller, C., Haug, G.T., Haug, C. & Hörnig, M.K. early view. A fossil aphidlion preserved together with its prey in 40 million-year-old Baltic amber. Palaeobiodiversity and Palaeoenvironments.
